# A New Thermosensitive *smc-3* Allele Reveals Involvement of Cohesin in Homologous Recombination in *C. elegans*

**DOI:** 10.1371/journal.pone.0024799

**Published:** 2011-09-21

**Authors:** Antoine Baudrimont, Alexandra Penkner, Alexander Woglar, Yasmine M. Mamnun, Margot Hulek, Cathrin Struck, Ralf Schnabel, Josef Loidl, Verena Jantsch

**Affiliations:** 1 Max F. Perutz Laboratories, Department of Chromosome Biology, University of Vienna, Vienna, Austria; 2 Department of Genetics, Technical University of Braunschweig, Braunschweig, Germany; Texas A&M University, United States of America

## Abstract

The cohesin complex is required for the cohesion of sister chromatids and for correct segregation during mitosis and meiosis. Crossover recombination, together with cohesion, is essential for the disjunction of homologous chromosomes during the first meiotic division. Cohesin has been implicated in facilitating recombinational repair of DNA lesions via the sister chromatid. Here, we made use of a new temperature-sensitive mutation in the *Caenorhabditis elegans* SMC-3 protein to study the role of cohesin in the repair of DNA double-strand breaks (DSBs) and hence in meiotic crossing over. We report that attenuation of cohesin was associated with extensive SPO-11–dependent chromosome fragmentation, which is representative of unrepaired DSBs. We also found that attenuated cohesin likely increased the number of DSBs and eliminated the need of MRE-11 and RAD-50 for DSB formation in *C. elegans*, which suggests a role for the MRN complex in making cohesin-loaded chromatin susceptible to meiotic DSBs. Notably, in spite of largely intact sister chromatid cohesion, backup DSB repair via the sister chromatid was mostly impaired. We also found that weakened cohesins affected mitotic repair of DSBs by homologous recombination, whereas NHEJ repair was not affected. Our data suggest that recombinational DNA repair makes higher demands on cohesins than does chromosome segregation.

## Introduction

The cohesin complex contains members of the highly conserved structural maintenance of chromosomes (SMC) protein family [1]. SMC proteins are involved in DNA condensation, cohesion, and repair. During mitosis, replicated sister chromatids are held together by cohesins in metaphase; this cohesion permits the bipolar orientation of the spindle kinetochores, allowing microtubules to separate the sister chromatids into the two daughter cells (for review see [2]). During meiosis, chromosomes face a new challenge: separation of the homologs during meiosis I to halve the ploidy of the cell. During metaphase I, cohesion supports the co-orientation of the kinetochores of bivalents (pairs of homologous chromosomes) [3]. This co-orientation leads to the separation of homologous chromosomes in anaphase I. Coordinated separation of homologous chromosomes and sister chromatids during meiosis I and II, respectively, is achieved by the two-step loss of cohesin from the arms and the centromeric regions [4].

Cohesin is a tetrameric complex composed of two SMC subunits, Smc1p (the worm homolog would be HIM-1) and Smc3p, and two non-SMC subunits, such as Scc1p and Scc3p in yeast [5]. SMC subunits bear nucleotide-binding domains (NBDs) at the amino and carboxy termini of linked long coiled-coil domains separated by a hinge domain. Each SMC protein folds on itself and forms a central region (coiled coil) with the hinge domain and the two NBDs at either end. Smc1p and Smc3p dimerize via the hinge domain, and the non-SMC subunits bind to the NBDs of the two SMCs. This ring is closed by the non-SMC subunit Scc1p, a member of the α-kleisin family [6]. Scc1 belongs to the mitotic specific cohesin complex, whereas during meiosis, cohesin complexes include the meiosis-specific kleisin Rec8 [1].

Homologs of the cohesin proteins have been identified in *C. elegans* [7], [8], [9], [10], [11]. In *C. elegans*, homozygous *smc-1* and *smc-3* deletion mutants cease their development at larval stages L1–L2, suggesting a maternal rescue of cohesins during the first stage of embryonic development [12]. Moreover, depletion of SMC-1 and SMC-3 by RNAi results in embryonic lethality with complete penetrance [9].

Cohesins delineate the axes of meiotic prophase chromosomes. These axes become the lateral elements of the synaptonemal complex (SC) [13]. In addition to the meiotic cohesin complex (SMC-1, SMC-3, REC-8, and SCC-3), *C. elegans* lateral elements contain HIM-3 and HTP-1, 2, and 3, which are related to budding yeast Hop1 [14], [15], [16] HTP-3 is required to load cohesins onto chromosomes during meiosis [7]. Additionally, HTP-3 is involved in the formation of DSBs [17].

Unlike in yeast, synapsis (i.e., the connection of axial elements by transversal filaments) is independent of the formation of DSBs in *C. elegans* [18]. However, as in other organisms, repair of DSBs entailing chiasmata takes place in the context of the SC [19]. Repair of DSBs requires the MRN complex to generate 5′ to 3′ resected DNA overhangs [20]. This resection is necessary to allow loading of the strand invasion protein RAD-51. As in yeast, in worms, the MRN complex is also needed for the formation of DSBs [21], [22]. However, under conditions of weakened REC-8 function, DSBs can be formed in the absence of RAD-50 or MRE-11 [23], which are components of the MRN complex. In meiosis, effective repair depends on the availability of a homologous chromosome as the template, because repair via the sister is inhibited by HIM-3, a constraint that is lost in late pachytene.

*C. elegans* hermaphrodite gonads are organized in a spatial gradient form distal to proximal representing consecutive stages of meiotic prophase I (leptotene/zygotene (transition zone, TZ), pachytene, diplotene, diakinesis) that follow the most distally positioned proliferative mitotic zone [13]. In leptotene, chromosomes condense and engage in the homolog search. During the zygotene stage, the SC starts to polymerize between paired homologs. After successful repair of the DSBs via the homolog in pachytene the chromosomes condense upon entry into diplotene and reach maximal condensation in diakinesis. In diakinesis the paired homologs (6 bivalents in *C. elegans*) are physically linked by one crossover.

Here, we report the isolation of a new thermosensitive *smc-3* allele with distinct reduced viability at the restrictive temperature (25°C). Our analysis revealed that *smc-3* mutant worms formed organized gonads, but they were defective in meiotic repair, with highly fragmented chromatin at diakinesis. We found significantly reduced cohesin complexes associated with chromatin and show that the homolog search process started with wild-type kinetics but because of defective synapsis, pairing could not be stabilized. Reduced amounts of cohesins rendered the chromatin more susceptible to meiotic DSB formation. We analyzed the repair defect observed in *smc-3* during meiosis by epistasis analysis. We also show that mitotic repair was impaired as soon as it relied on homologous recombination. Our results demonstrate the essential role of cohesin in mitosis and meiosis separate from its role in cohesion, the latter requires less cohesins than the repair of DSBs.

## Results

### *t2553*: a new temperature-sensitive allele of the cohesin subunit *smc-3*

**Figure 1:**
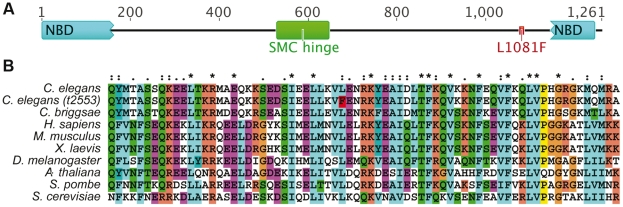
The new allele *smc-3 (t2553)*. (A) Domain organization of *C. elegans* SMC-3 highlighting the position of the L1081F amino acid change; domain organizations predicted by CDART [45]. (B) Alignment of SMC-3 sequences for the indicated organisms highlights conservation of the mutated leucine (highlighted in red in *t2553*).

*t2553* was isolated as a temperature-sensitive maternal effect lethal mutant. Mapping and complementation tests confirmed *t2553* as a new allele of *C. elegans smc-3*. Sequencing of the locus revealed a point mutation (a C-to-T transition) in the coding sequence of *smc-3* at position 3241, resulting in a leucine (L)-to-phenylalanine (F) amino acid change at position 1081. The L1081F mutation resides in a coiled-coil region in proximity to the C-terminal ATPase domain (Figure 1A). Alignment of SMC-3 proteins from various phyla revealed that this leucine is widely conserved (Figure 1B).

*smc-3* mutants displayed a reduced brood size compared to wild type when grown at 16°C (Table 1). A total of 89±3% of the *smc-3* embryos hatched (wild type: 98±3%), and 4±2% of the viable offspring were males (wild type: 0%). Both the reduced brood size and the documented Him (*h*igh *i*ncidence of *m*ales) phenotype are consistent with a chromosome segregation failure and a putative meiotic defect in *smc-3* mutants. Shifting L1 larvae to 25°C for approximately 55 h reduced the brood size even further (Table 1) with a high embryonic lethality (hatch rate: *smc-3*, 2±2%; wild type: 98±3%). No obvious growth or morphological defects were observed in the surviving progeny. This particular *smc-3* allele therefore allowed us to analyze the role of SMC-3 in meiotic chromosome behavior.

**Table 1:** Brood size and hatch rates

	16°C		25°C	
	brood size	hatch rate	brood size	hatch rate
wild type	258±25 eggs^a^	98±1%	187±20 eggs^a^	98±2%
*smc-3*	188±38 eggs^a^^,^ b	89±3%^a^^,^ b	81±21 eggs^a^^,^ b	2±2%^a^^,^ b

a Student's *t*-test, *p*<0.05 comparing genotypes at 16°C and 25°C.

b Student's *t*-test, *p*<0.05 comparing *smc-3* to the wild type at respective temperature.

Brood size and hatch rate of wild-type and *smc-3* mutant worms at 16°C and 25°C (mean±SD). Progeny of seven worms were scored.

### Repair of programmed DSBs impaired in *smc-3*

**Figure 2:**
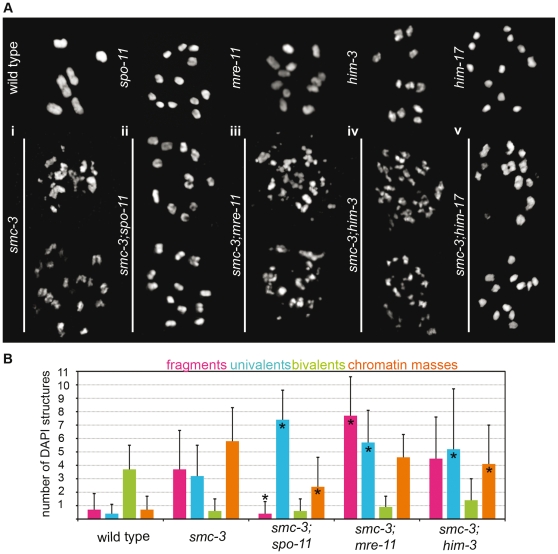
Fragmentation at diakinesis resulting from defective DSBs repair. (A) Representative DAPI structures found at diakinesis in: (i) top: wild type, bottom: *smc-3*; (ii) top: *spo-11*, bottom: *smc-3; spo-11*; (iii) top: *mre-11,* bottom: *smc-3;mre-11;* (iv) top: *him-3*, bottom: *smc-3;him-3*; and (v) top: *him-17*, bottom: *smc-3;him-17.* (B) Quantification of DNA fragmentation at last diakinesis. Classes (fragments, univalents, bivalents, and DNA masses) were defined by the volume (v) and the “sphericity” (s) of the DAPI structures at the last diakinesis before the spermatheca (see Materials and Methods). Single star indicates significant differences (*p*<0.05, Student's *t*-test) for fragments, univalents, bivalents, and masses between double mutants and the *smc-3* single mutant. Error bars represent SD.

Chromatin masses that varied in numbers and size could be seen instead of the normal six DAPI-stained bodies in *smc-3* diakinesis (Figure 2Ai). The numerous small chromatin structures in diakinesis nuclei of *smc-3* hermaphrodites were indicative of DNA fragmentation, which is also observed in DNA repair–deficient mutants [10]. Depleting the SPO-11 endonuclease in *smc-3* mutants suppressed the formation of chromatin fragments in diakinesis (Figure 2Aii, bottom). In 49 diakinesis nuclei of *spo-11;smc-3* double mutants, 12 DAPI-positive structures could be seen on average (standard deviation [SD]: 0.2). Smaller chromatin fragments were completely absent. We therefore conclude that the fragmentation observed at diakinesis in *smc-3* mutants originated from defective meiotic crossing over resulting from SPO-11–induced DSBs.

### Requirement of the MRN complex for DBS formation bypassed in *smc-3* mutants

In *C. elegans*, MRE-11 is required for both formation and repair of DSBs [21], [22]. In the double mutant *smc-3;mre-11,* we would therefore expect 12 univalents, as was seen in the *smc-3;spo-11* double mutant. However, we observed massive fragmentation at diakinesis (Figure 2Aiii, bottom).

Because both the *smc-3* single mutant and the *smc-3;mre-11* double mutant displayed repair defects, we sought to quantify the fragmentation observed at diakinesis. To avoid artifacts generated by projections of pictures, this quantification was done on picture stacks measuring the volume and the “sphericity” of the DAPI structures (see Materials and Methods). In contrast to the wild type, where 3.7±1.8 (mean±SD) DAPI structures represented connected bivalents (Figure 2Ai, top and 2B, *n* = 15 diakinesis), quantification showed that chromosome fragments (3.7±2.9, *n* = 14 diakinesis) and “chromatin masses” (5.8±2.5 SD, n = 14 diakinesis) were the most prevalent classes of DAPI structures in *smc-3* mutants (Figure 2B).

Using this method, we detected an overall number of 10.9±0.9 (*n* = 18 diakinesis) DAPI structures and on average 7.4±2.2 (*n* = 18 diakinesis) univalents (Figure 2B) in *smc-3;spo-11* double mutants. Quantification confirmed that fragmentation was increased in *smc-3;mre-11* compared to *smc-3* worms (Figure 2B). In *smc-3;mre-11* worms, the number of fragments and univalents was significantly increased compared to the *smc-3* single mutant (Student's *t*-test, *p*<0.05). The *smc-3;rad-50* double mutant likewise displayed an increase in fragmentation (unpublished data).

These results indicate that the MRN complex was dispensable for the formation of DSBs in *smc-3* mutants.

### Fewer cohesin complexes on *smc-3* chromosomes not associated with defective cohesion

**Figure 3:**
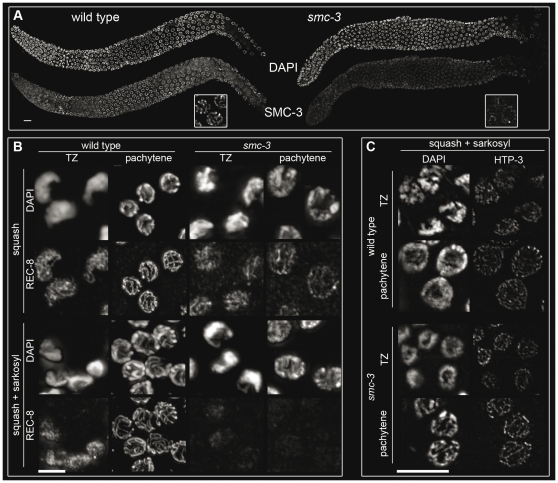
Chromosomes axes are impaired in *smc-3.* (A) Immunostaining of SMC-3 in wild-type and *smc-3* worms. (B) Squashed nuclei untreated and washed with sarkosyl in wild-type and *smc-3* worms in the TZ and pachytene stained with anti-REC-8 antibody. (C) Squashed nuclei washed with sarkosyl stained with anti-HTP-3 in wild-type and *smc-3* worms in TZ and pachytene. Bar: 10 µm.

We compared loading of SMC-3 onto chromosome axes in *C. elegans smc-3* and wild-type worms. In wild-type worms, SMC-3 delineated the chromosome axes, whereas in *smc-3*, only short and weakened stretches were observed (Figure 3A). The reduced amounts of SMC-3 could result either from reduced loading or reduced stability of the complex; notably, we did not see a gradual decrease in the SMC-3 signal during meiotic progression. To discriminate between these two possibilities, we examined the abundance of REC-8 in squashed nuclei with stringent sarkosyl washes. (Figure 3B). We reasoned that the addition of detergent should not further decrease the REC-8 signal if cohesin loading was already reduced in the mutant. However, the addition of detergent led to a strong reduction in REC-8 levels in *smc-3* mutants (Figure 3B), suggesting that the point mutation in the coiled-coil domain impaired the stability of the cohesin complex on chromatin. However, this assay cannot judge on the amount of cohesin loaded in the mutant.

To test the validity of the detergent assay, we also probed for HTP-3. Proper loading of the cohesin complex requires efficient HTP-3 loading [7]. The detergent washes did not remove HTP-3 from either wild-type or *smc-3* mutant squashed nuclei. (Figure 3C), confirming the validity of the approach. Furthermore, we subjected *syp-2* squashed nuclei to the same assays (REC-8 and HTP-3 staining with and without sarkosyl). Deletion of SYP-2, a central element component of the SC, results in unsynapsed chromosomes [19]. We did not observe a decrease in REC-8 or HTP-3 signals after washing *syp-2* mutants with sarkosyl (Figure S1B). This reinforces the specificity of the assay, because a lack of synapsis could not account for the decrease in REC-8 loading that we observed after washing with sarkosyl. Similarly, no defects in SMC-3 loading were detectable after depletion of SYP-2 (Figure S1A).

Altogether, these results reinforce the idea that the L1081F mutation in *smc-3* leads to unstable chromatin-associated cohesin complexes entailing a reduced amount of cohesin complexes on chromatin. The presence of 12 univalents instead of 24 sister chromatids in *spo-11;smc-3* diakinesis (Figure 2Aii, bottom) suggests intact cohesion with this particular allele.

To test this assumption, cohesin loading was further reduced by RNAi-mediated depletion of the cohesin subunit REC-8. Indeed, diakinesis nuclei of *spo-11;smc-3;rec-8(RNAi)* triple mutants always showed more than 20 signals (in all 26 diakinesis nuclei scored from eight independent gonads), consistent with the expected 24 isolated sister chromatids in cohesion-deficient mutants (Figure S2). *spo-11;rec-8(RNAi)* control worms also displayed more than 20 DAPI-positive structures (unpublished data). The reduced amount of cohesins appeared to be sufficient for the establishment of cohesion but insufficient for DSB repair in *smc-3*. Additionally, when pairing of the pairing center (PC) protein HIM-8 was assayed, more than two HIM-8 signals were detected in the mitotic zone of *smc-3* gonads only rarely, indicating that cohesion in most cells is effective (Figure S3). In contrast, during the meiotic time course homologous associations were unstable which can be explained by the failure to establish a proper SC (text S1 and Figures S4 and 5). However homologous pairing prior to SC formation was normal in the mutant.

### *smc-3* mutants deficient in early steps of meiotic DSB repair

**Figure 4:**
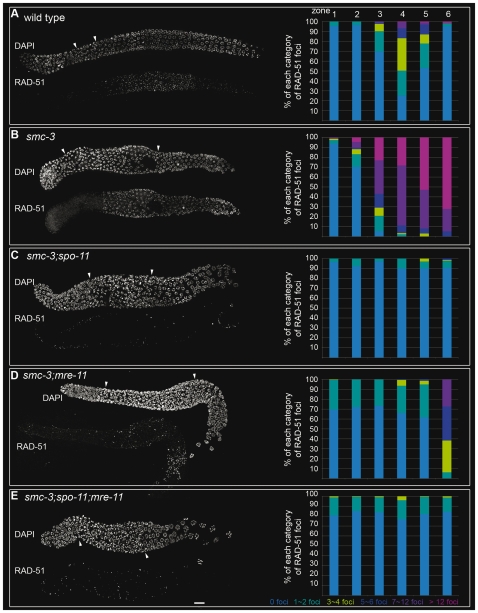
*smc-3* mutants are defective in repair of meiotic DSBs. Left, immunostaining of RAD-51. Right, quantification of RAD-51 foci in wild-type (A), *smc-3 (tm2553)* (B), *smc-3;spo-11* (C), *smc-3;mre-11* (D). and *smc-3;spo-11; mre-11* (E) worms. Gonads were divided into six zones of equal length for quantification. Arrowheads indicate the zone with clustered chromatin. Bar: 10 µm.

To study meiotic DNA repair, we stained nuclei for the strand-invasion protein RAD-51 [24]. Gonads were divided into six zones of equal lengths, and the number of RAD-51 foci were counted per nucleus. In wild-type worms, RAD-51 foci were observed from early pachytene until mid-pachytene (Figure 4A, left) with a maximum number of 7–12 RAD-51 foci in zone 4 (Figure 4A, right). In *smc-3* mutants, the first RAD-51 signals were seen earlier (Figure 4B, left). The number of RAD-51 foci rose from entry into meiosis (zone 2) until late pachytene (zone 6) and continued to accumulate (>12 foci in 70% of nuclei in zone 6; Figure 4B, right), in fact the frequency of foci increased faster in *smc-3*. Few RAD-51 foci could be detected at diakinesis (unpublished data). These data suggest that early steps of HR require functional cohesins and that more DSBs may be formed in *smc-3* mutants.

Next, we wanted to confirm that RAD-51 foci accumulation was SPO-11 dependent. Depletion of SPO-11 in *smc-3* decreased RAD-51 signals (Figure 4C, left). Indeed, in the double mutant *smc-3;spo-11*, a maximum of one or two RAD-51 foci could be observed during the time course (Figure 4C, right). When we scored loading of RAD-51 foci in *smc-3;mre-11* double mutants (Figure 4D, left), we found that until late pachytene (before zone 6), only the class of nuclei with one or two RAD-51 foci was observed. Strikingly, in late pachytene, a significant increase in the number of RAD-51 foci for all classes was found, with a maximum of about 7–12 foci (Figure 4D, right). *smc-3;rad-50(ok197)* double mutants displayed the same phenotype (unpublished data).

We next tested whether this appearance of RAD-51 foci in *smc-3;mre-11* mutants in late pachytene depended on breaks introduced by SPO-11. In the triple mutant *smc-3;spo-11;mre-11*, the number of nuclei with 1–2 RAD-51 foci was significantly decreased compared to *smc-3;mre-11* mutants (Fisher's exact test per class and per zone, *p*<0.05). Remarkably, we did not observe an increase in the number of RAD-51 foci during the time course (Figure 4E, right), confirming that the appearance of excess RAD-51 foci in *smc-3;mre-11* mutants was due to breaks introduced by SPO-11. Furthermore, in *smc-3;spo-11;mre-11* mutants, chromosome fragmentation at diakinesis was absent (Figure S6).

The results of our analysis confirm previous reports (using different components of the protein complexes involved) that the MRN complex is required to restrain the inhibitory action of cohesins on DSB induction [23]. In addition, weakened cohesion led to an increased number of detectable RAD-51 foci, likely due to more DSB induction, suggesting that intact chromosome axes restrain the action of the DSB break machinery.

### Repair via the sister chromatid is impaired in *smc-3*

Next, we tested the effectiveness of DSB repair via the sister chromatid. HIM-3 is a major constituent of the lateral element of the SC and exerts an inhibitory effect on meiotic DSB repair via the sister chromatid [14], [15]. Fragmentation at diakinesis was still present in the double mutant *smc-3;him-3* (Figure 2Aiv, bottom), suggesting that repair via the sister chromatid was not taking place efficiently. Quantification of the DAPI signals revealed a shift to “univalents” from “chromatin masses” (Student's *t*-test, *p*<0.05) suggesting that some repair might still be taking place (Figure 2B). Therefore, we concluded that despite effective sister chromatid cohesion in *smc-3* mutants, repair of DSBs via the sister chromatid was impaired.

### Can weakened cohesion lead to reversion of DSB-refractory chromatin?

The dramatic fragmentation at diakinesis in *smc-3* mutants could reflect a defect in the repair of DSBs, result from an open chromatin conformation that is more permissive to formation of DSBs, or both. The early accumulation of RAD-51 in *smc-3* lends support to the second argument. Previously, it was shown that a mutation in the condensing component DYP-28 could “re-open” chromatin during DSB-refractory chromatin states in *him-17* mutants [25]. Therefore, we compared our *smc-3* mutant to *dpy-28* worms with respect to DSB induction in the DSB compromised mutant *him-17*. Depleting HIM-17 in *smc-3* resulted in diakinesis similar to that seen in *smc-3;spo-11* double mutants, with mostly univalents and reduced fragmentation (Figure 2Av, bottom). This suggests that in contrast to compromised condensin complexes, weakened cohesion could not reverse a failure to induce DSBs in *him-17* mutants.

### *smc-3* mutation affects mitotic HR but not NHEJ repair

**Figure 5:**
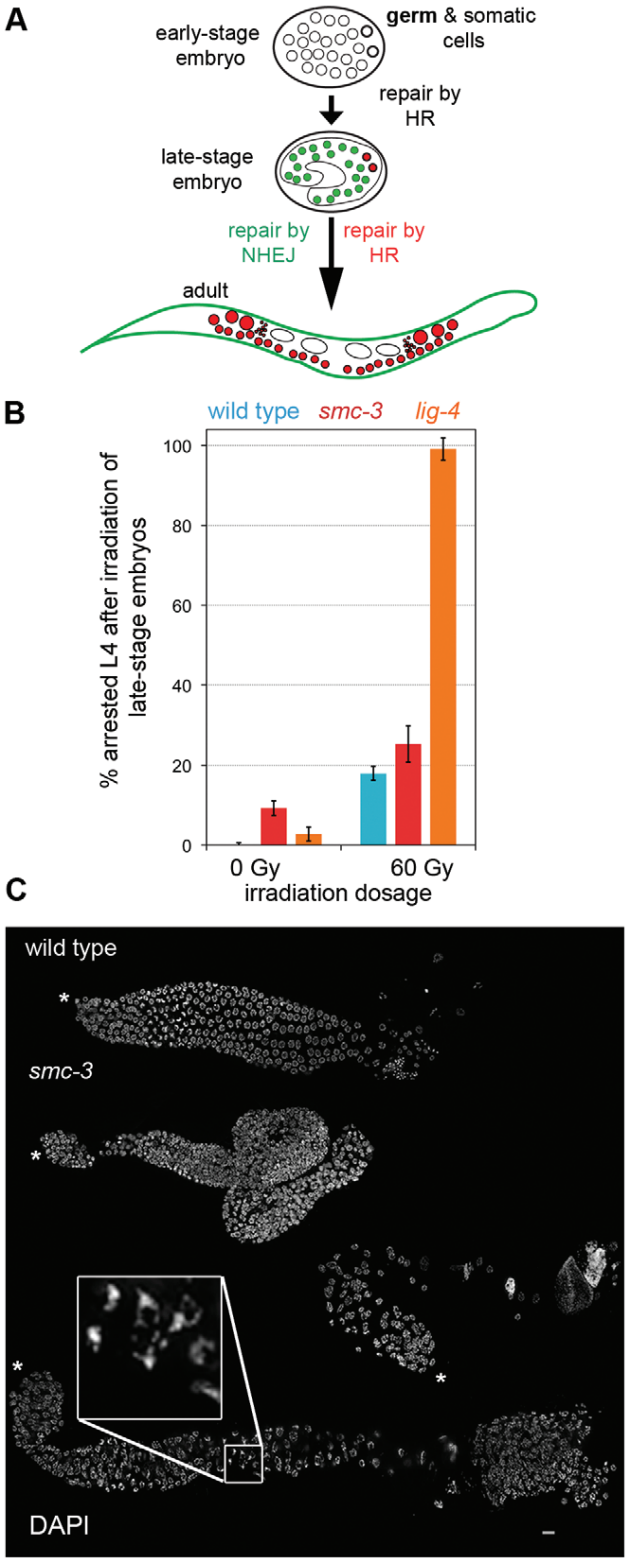
The *t2553* allele impairs mitotic HR repair but not NHEJ repair. (A) Schematic drawing of an embryo with germ (bold circle) and somatic cells (circle). Green color highlights cells repairing DSBs by NHEJ; red cells repair via HR. In the adult, small red circles represent spermatozoa and open ovals represent embryos. (B) Percentage of arrested L4 66 h after 60 Gy irradiation of late-stage embryos with the indicated genotypes. Error bars represent standard error of the mean (SEM). (C) DAPI staining of wild-type and *smc-3* gonads released from worms irradiated (60 Gy) as late-stage embryos. Stars mark the distal tip of the gonad. Bar: 10 µm.

*C. elegans* genetics is a powerful tool that can be used to separately study two different mitotic repair pathways (Figure 5A). Early embryos repair DSBs by homologous recombination (HR), whereas late-stage embryos repair via the nonhomologous end joining (NHEJ) repair pathway [26]. In addition, repair of DSBs via HR takes place in the primordial germ cells Z2 and Z3 during larval development of the worm (Figure 5A).

Irradiation of late-stage embryos allowed us to test the proficiency of the NHEJ repair pathway using a dose of 60 Gy. *lig-4* mutants, which are defective in the NHEJ repair pathway, slowed down their development when irradiated with 60 Gy as late-stage embryos: all of the worms were still at the L4 stage 66 h after irradiation (Figure 5B). This was in contrast to wild-type worms and *smc-3* mutants, in which only 19% and 24%, respectively, of arrested worms were at the L4 stage 66 h after irradiation (Figure 5B). We therefore concluded that *smc-3* mutants were proficient in the somatic NHEJ repair pathway.

To assess the proficiency of the HR occurring in Z2 and Z3 cells, gonads of adult wild-type and *smc-3* worms that were irradiated (60 Gy) as late-stage embryos were released. DAPI-stained *smc-3* gonads were disorganized, and some showed a lack of meiotic entry (absence of a transition zone) and lacked mature sperm, supporting the idea that mitotic HR repair was also defective (Figure 5, inset) Surprisingly, cells with nuclei of different sizes, indicative of chromosomal nondisjunction, were absent, reinforcing the idea that a reduced amount of cohesin was sufficient for proper segregation of mitotic chromosomes.

### DNA damage checkpoint is operating in *smc-3*

Intrigued by the large number of nuclei with more than 12 RAD-51 foci in the last zone of *smc-3* gonads (Figure 4B, left), we first assayed apoptosis in *smc-3* mutants. Acridine orange staining revealed that apoptosis was significantly increased in *smc-3* worms, with 7.8±2.1 (mean±SD) apoptotic corpses (*n* = 36 gonads), compared to wild-type worms, with 3.7±1.3 apoptotic corpses (*n* = 31 gonads).

**Figure 6:**
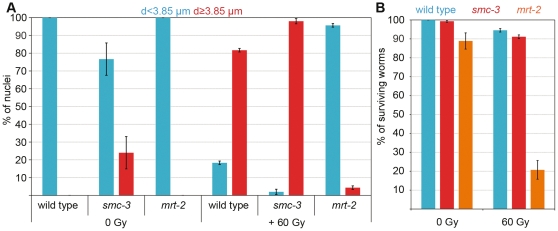
DNA damage checkpoints are working in *smc-3*. Proportion of nuclei with “normal” (<3.85 µm) or “double” (≥3.85 µm) diameters before and 8 h after 60 Gy irradiation (more than 200 nuclei assayed in five different gonads per genotype for the indicated genotypes (N2, *smc-3*, and *mrt-2*). Error bars represent SEM. (B) Percentage of surviving worms 48 h after 60 Gy irradiation. Error bars represent SEM. More than 500 L1s were scored per genotype.

We next asked whether the DNA damage checkpoint was properly activated. Triggering the checkpoint in the mitotic compartment leads to enlargement of nuclei [27]; therefore, we fixed and DAPI stained gonads and then counted nuclei with increased diameters within the first 50 µms from the distal tip of the gonad. In *mrt-2* mutants, which are deficient in DNA damage signaling [28], there was only a slight increase in nuclei diameter 8 h after 60 Gy irradiation (0 Gy: 3.03±0.3 µm [mean± SD; *n* = 25 nuclei], 60 Gy: 3.5±0.3 µm [*n* = 23 nuclei]) compared to wild type (0 Gy: 3.03±0.3 µm [*n* = 28 nuclei], 60 Gy: 5.7±0.9 µm [*n* = 30 nuclei]). The average size of *mrt-2* nuclei plus the SD was used to define the threshold of checkpoint activation. The analysis revealed that even without irradiation challenge (0 Gy), a significant number of cells in the mitotic portion of *smc-3* gonads had activated the DNA damage checkpoint (Figure 6A), whereas no activation of the checkpoint was observed in wild-type or *mrt-2* worms. We cannot exclude the possibility that the increased number of enlarged nuclei in *smc-3* mutants before irradiation might also be triggered by the activation of the mitotic spindle checkpoint due to a slight cohesion or chromosome alignment defect. Moreover, 8 h after irradiation, the proportion of cells activating the DNA damage checkpoint in *smc-3* mutants was even larger than in wild type (Figure 6A). Therefore, the DNA damage checkpoint was properly activated in *smc-3* worms.

To test if the DNA damage checkpoint was also properly activated during the development of the worms, we performed the L1 assay as described in [29]. *C. elegans* develops through four larval stages (L1–L4). Worms deficient for the DNA damage checkpoint do rarely survive to the adult stage upon irradiation. Irradiation with 60 Gy had almost no impact on the development of *smc-3* worms into adults, the same as for wild-type worms; however, only 20% of *mrt-2* worms reached the adult stage (Figure 6B). This supports our conclusion that during worm development, the DNA damage checkpoint was properly activated in *smc-3* worms.

## Discussion

We isolated a conditional partial loss-of-function mutant of the cohesin component SMC-3 and showed that a reduction in cohesin molecules mainly impaired repair of DSBs in mitosis and meiosis. The mutation (L1081F) in the newly identified thermosensitive allele *smc-3(t2553)* resides in the coiled-coil region close to the NBDs. In human Hela cells, Smc1 is phosphorylated in the coiled-coil region (serines 957 and 966) after induction of DSBs in an ATM-dependent manner [30], [31]. Two similar sites of phosphorylation (serines 1067 and 1083) are found in the coiled-coil region close to the NBDs of Smc3. Recently, phosphorylation of Smc3 at serine 1083 was shown to take place in response to DNA damage in Hela cells [32]. In the *t2553* allele, the mutation L1081F might interfere with phosphorylation of SMC-3 upon DNA damage signaling, in addition to the overall reduction in stably DNA bound cohesin molecules that could contribute to the observed DNA repair defects.

From our analysis, it appears that the mutation introduced in the *t2553* allele (L1081F) led to reduced stability of the cohesin complexes on chromosome arms during meiosis, as shown by a weaker SMC-3 signal in immunofluorescence assays. In addition, the cohesin component REC-8 could be expelled from chromatin by detergent treatment of the *smc-3* mutant. This suggests that stable association of cohesin with chromatin was reduced, but sister chromatid cohesion remained robust. Indeed, *smc-3* mutants formed organized gonads at the restrictive temperature (25°C) and segregation defects were only rarely detectable in the mitotic zone in *smc-3* gonads. In addition, in *smc-3;spo-11* worms, 12 DAPI structures were observed at diakinesis, whereas 24 DAPI structures were observed after REC-8 RNAi treatment in this double mutant. This *smc-3* mutant, which lacks a major subunit of the cohesin complex, demonstrates that even a considerable reduction in the number of cohesin molecules on chromatin does not notably affect cohesion in either mitosis or meiosis in *C. elegans*. This is in agreement with a recent report that reducing functional cohesin rings by 87% does not notably affect cohesion in yeast [33].

Nevertheless, this *smc-3* mutant displays defects in meiotic and radiation-induced mitotic DSB repair. It is known that cohesion supports DSB repair, presumably by connecting the damaged site to the sister template in mitosis [34], and cohesin complexes are recruited to DSB-flanking regions extending up to several kilobases [34]. The reduced amount of stably chromatin-bound cohesin complexes in the *smc-3* mutant could explain the sensitivity to DNA damage that we observed during mitosis. Indeed, a sufficient amount of cohesin might not be established at the site of the DSBs.

It has been proposed that cohesins inhibit repair via the homologous chromosome [35]. In fact, holding sister chromatids together in meiosis might be unproductive when DSB-carrying DNA strands should detach from their sisters in order to undergo recombination with the homolog [36]. Therefore, in meiosis, the requirement for cohesin might be different, and might rather be required to create the platform for lateral element assembly as the backbone of the synaptonemal complex [5], [10], [37]. The dramatic increase in RAD-51 signals and their persistence until later stages of meiotic prophase I in *smc-3* argue for a defect in DSB repair downstream of the loading of RAD-51. We found that weakened SMC-3 did not allow DSB formation in the absence of HIM-17, but did in the absence of MRE-11/RAD-50. Therefore, the role of cohesion in supporting DSB generation is distinct from that of HIM-17, which confers competence for meiotic DSB formation by methylation of histone H3 [38]. This demonstrates that the MRN complex and HIM-17 act in different pathways upstream of SPO-11.

In the double mutant *smc-3*;*mre-11*, we observed pachytene chromosome fragmentation and increased RAD-51 foci as hallmarks of DSBs. In the absence of MRE-11 or RAD-50, DSBs are not observed [21], [23]. It should be noted that the dependence on *rad-50* for DSB formation is also partially abrogated in *htp-1* and *him-3* mutants, both of which are defective in functions connected to axial elements [23]. These mutants are most likely altered in composition or organization of chromosome axes, thereby making the requirement for the MRN complex dispensable for DSB formation. Weakened SMC-3 suspends the requirement for MRE-11 (or MRN) for DSB formation, as does weakened REC-8. Because both SMC-3 and REC-8 are components of meiotic cohesin, it is likely that weakening the cohesin complex as a whole promotes the generation of DSBs by SPO-11. It is conceivable that the chromosome axis structure interferes with the access or activity of SPO-11 and that this obstacle is locally and/or temporally released by the activity of MRN. This could represent yet another layer in the control of meiotic recombination. Notably, we observed increased RAD-51 signals right after meiotic entry, suggesting that weakened cohesion augments DSB induction.

Smc3 is required for the complete activation of damage signaling to checkpoints upon introduction of artificial DSBs in human cells [32] involving the cohesin complex in the activation of the DNA damage checkpoint. We can state with certainty that the NHEJ repair pathway in mitotic divisions still takes place in the weakened cohesion mutant *smc-3*. Although repair of meiotic lesions was impaired in worms with the *t2553* allele, we found that the few cohesion molecules left were nonetheless able to activate DNA damage checkpoints effectively.

To summarize, this work reinforces the idea that effective repair of DSBs during mitosis and meiosis is more vulnerable to loss of cohesin complexes compared to proper chromosome segregation. It also confirms that, even for holocentric chromosomes, few cohesin complexes are required for proper segregation. Furthermore, the newly isolated temperature-sensitive *smc-3* allele represents a new tool with which to study the effects of weakened cohesion in worms.

## Materials and Methods

### Nematode strains, strain construction and culture conditions

All *C. elegans* strains were cultured using standard techniques (Brenner, 1974). The following *C. elegans* strains were used: N2 Bristol, GE4345 *smc-3(t2553)* AV276 *syp-2(ok307)* [19], AV106 *spo-11(ok79)* [18], AV112 *mre-11(ok179)* [21], VC418 *him-3(gk149)* [15], VC255 *him-17(ok424)* [38], CB5348 *mrt-2(e2663)* [28]. Nematode strains were provided by the *Caenorhabditis Genetics Center*, which is funded by the NIH National Center for Research Resources (NCRR).

### Cytological preparation of gonads and immunostaining

Hermaphrodite gonads were dissected and fixed as described in [39]. For chromatin staining, the preparations were mounted in Vectashield anti-fade (Vector Laboratories Inc., Burlingame, CA) containing 2 µg/ml 4′6-diamidino-2-phenylindole (DAPI). For immunostaining, gonads were blocked in 3% BSA/1x PBS for 20 min. Primary antibodies were applied overnight at 4°C. Antibodies were diluted in 1x PBS/0.01% sodium azide as follows: anti-SUN-1 Ser8-Pi [40] 1∶700, anti-HIM-8 [41] 1∶500, anti-ZIM-3 [42] 1∶100, anti-RAD-51 [24] 1∶300, anti-SYP-1 [43] 1∶200, anti-HTP-3 [17] 1∶500, anti-SMC-3 [44] 1∶10. After 3 washes in 1x PBST (1x PBS, 0.1% Tween-20), secondary antibodies were applied for 2 h at RT. After washes in PBST, samples were mounted.

### Fluorescence *in situ* hybridization (FISH)

The PCR-amplified 5S rDNA was used as a probe for the right arm of chromosome V. The 5S rDNA was labeled by PCR with digoxigenin-11-dUTP. FISH was performed as described in [10]. Hybridized digoxigenin-labeled probes were detected with FITC-conjugated anti-digoxigenin antibodies (1∶100). Slides were mounted in Vectashield/DAPI.

### Microscopy and evaluation

A Zeiss Axioskop epifluorescence microscope was used and images were recorded with a cooled CCD camera (Photometrics Ltd., Tucson, AZ). Evaluation of cytological phenotypes was performed in animals shifted to 25°C at the L1 stage for 66 hours. 3D stacks of images were taken (MetaVue software, Universal Imaging Co., Downingtown, PA), deconvolved (AutoDeblur software, AutoQuant Imaging Inc., Troy, NY) and projected (Helicon Focus software http://helicon.com.ua/heliconfocus/). Artificial coloring and merging were undertaken with Adobe Photoshop 7.0 software (Adobe Systems Incorporated).

### RNA interference of *rec-8*

Double stranded RNA was produced by *in vitro* transcription and injected after [10].

### Mitotic repair assay

Ten young adult worms for each genotype (wild type, *smc-3, mrt-2*) were allowed to lay eggs at the restrictive (25°C) for 2 h. Next, hermaphrodites were removed from the plates and after 3∼4 h at 25°C the eggs were γ-irradiated with a dose of 60 Gy using a ^137^Cs source. Synchrony of the late-stage embryo at the bean stage was controlled before irradiation. Worms were kept at the restrictive temperature for 66 hours and then the number of arrested worms was scored. The L1 assay was performed as described in [29].

### Quantification of DNA fragmentation

Stack pictures from diakinesis were deconvolved (AutoDeblur software, AutoQuant Imaging Inc., Troy, NY). Volumes were quantified with the 3D Object Counter plugin using ImageJ. Classes were defined by the volume (v) and the sphericity (s) of the DAPI structures at the last diakinesis before the spermatheca. Sphericity, as a measure of the roundness of an object, is the ratio of the surface area of a sphere (with the same volume as the given object) to the surface area of the object. Using this method bivalents and univalents could be identified (bivalents: v = 3.0±1.1 µm^3^ (mean±SD), s = 0.65±0.07 (mean±SD), n = 15 diakinesis; univalents: v = 1.2±0.4 µm^3^, s = 0.80±0.06, SD, n = 16 diakinesis). DAPI structures with a larger volume than the defined volume of bivalents or with a volume in the range of bivalents but with different sphericity than bivalents were classified as “chromatin masses”. DAPI structures with a smaller volume than a univalent or with a volume of a univalent but different sphericity were defined as fragments. 3-dimensional projections of deconvolved pictures and counted volumes are provided for each genotype (Video S1, S2, S3, S4, S5 and S6).

## Supporting Information

Figure S1 **Lack of synapsis cannot account for a decrease in immunostaining after washing with sarkosyl.** A. Immunostaining of SMC-3 in *syp-2* mutant worms. B. *syp-2* squashed nuclei untreated and washed with sarkosyl stained with anti-REC-8 in TZ and pachytene. C. *syp-2* squashed nuclei washed with sarkosyl stained with anit-HTP-3 in TZ and pachytene. Bar: 10 µm.
(TIFF)

Figure S2 **Meiotic cohesion is effective in** ***smc-3*** **.** Representative diakinesis of indicated genotypes (wild type, *spo-11, smc-3, smc-3;spo-11, smc-3;spo-11;rec-8*(RNAi).
(TIFF)

Figure S3 **Rare mitotic defects in** ***smc-3*** **mutants worms.** Time course of HIM-8 pairing in wild type and *smc-3* revealed the presence of 3 foci in the mitotic zone of *smc-3* on rare occasions.
(TIFF)

Figure S4 **Proper loading of the PC protein ZIM-3 in** ***smc-3*** **but defective synapsis.** A. Immunostaining of the pairing center protein ZIM-3 in wild type and *smc-3* (DAPI blue). B. Immunostaining of SUN-1S8Pi in wild type and *smc-3;* (DAPI blue). C. Time course for pairing of HIM-8 in wild type and *smc-3*. Gonads were subdivided into 6 zones of equal lengths. D. Time course for pairing with the 5S rDNA FISH probe (chromosome V) in wild type and *smc-3*. Gonads were subdivided into 6 zones of equal lengths. E. Immunostaining of SYP-1 in wild type and *smc-3*; pachytene nuclei enlarged in the inset; bar 10 µm.
(TIFF)

Figure S5 **HIM-3 loading is strongly reduced in** ***smc-3*** **.** Immunostaining of HIM-3 (red) in wild type and *smc-3*; bar 10 µm.
(TIFF)

Figure S6 **Absence of fragmentation in the triple mutant** ***smc-3;spo-11;mre-11*** **.** A. Representative diakinesis of the indicated genotypes (wild type, *smc-3, smc-3;mre-11, smc-3;mre-11;spo-11*). Bar 5 µm. B. Quantification of DAPI structures at diakinesis in *smc-3;spo-11;mre-11*.
(TIFF)

Video S1 3-dimensional projection of deconvolved stack pictures (left) and colored surface of the DAPI structures (right) in wild type.
(AVI)

Video S2 3-dimensional projection of deconvolved stack pictures (left) and colored surface of the DAPI structures (right) in *smc-3*.
(AVI)

Video S3 3-dimensional projection of deconvolved stack pictures (left) and colored surface of the DAPI structures (right) in *smc-3; spo-11*.
(AVI)

Video S4 3-dimensional projection of deconvolved stack pictures (left) and colored surface of the DAPI structures (right) in *smc-3; mre-11*.
(AVI)

Video S5 3-dimensional projection of deconvolved stack pictures (left) and colored surface of the DAPI structures (right) in *smc-3; him-3*.
(AVI)

Video S6 3-dimensional projection of deconvolved stack pictures (left) and colored surface of the DAPI structures (right) in *smc-3; him-17*.
(AVI)

Text S1 (DOC)
