# Supplementary figures and images for: A New Thermosensitive smc-3 Allele Reveals Involvement of Cohesin in Homologous Recombination in C. elegans
Source: PLoS One. 2011 Sep 21;6(9):e24799. doi: 10.1371/journal.pone.0024799 (PMC3177864; doi:10.1371/journal.pone.0024799)

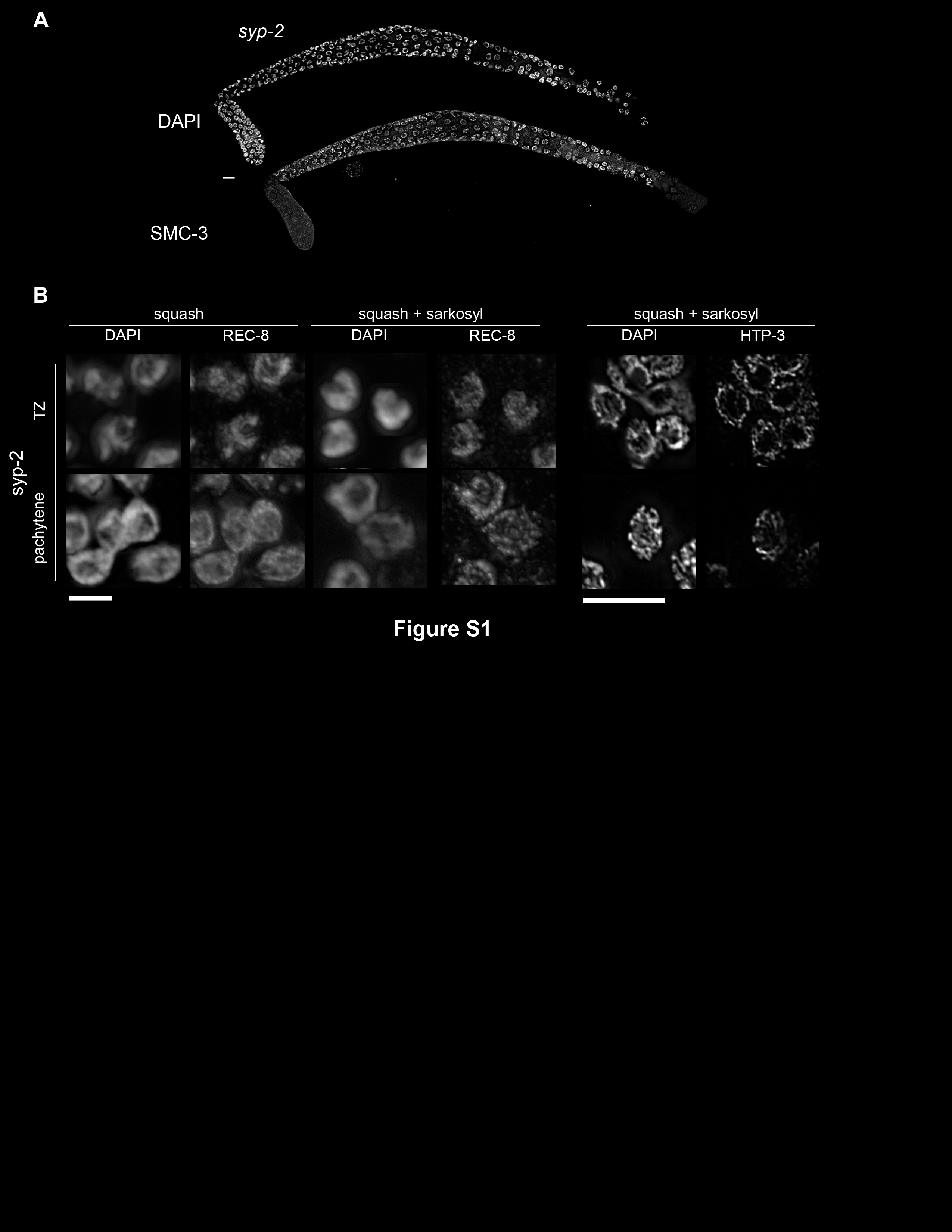

Supplement: Figure S1 — Lack of synapsis cannot account for a decrease in immunostaining after washing with sarkosyl. A. Immunostaining of SMC-3 in syp-2 mutant worms. B. syp-2 squashed nuclei untreated and washed with sarkosyl stained with anti-REC-8 in TZ and pachytene. C. syp-2 squashed nuclei washed with sarkosyl stained with anit-HTP-3 in TZ and pachytene. Bar: 10 µm. (TIFF) [file pone.0024799.s001.tiff]

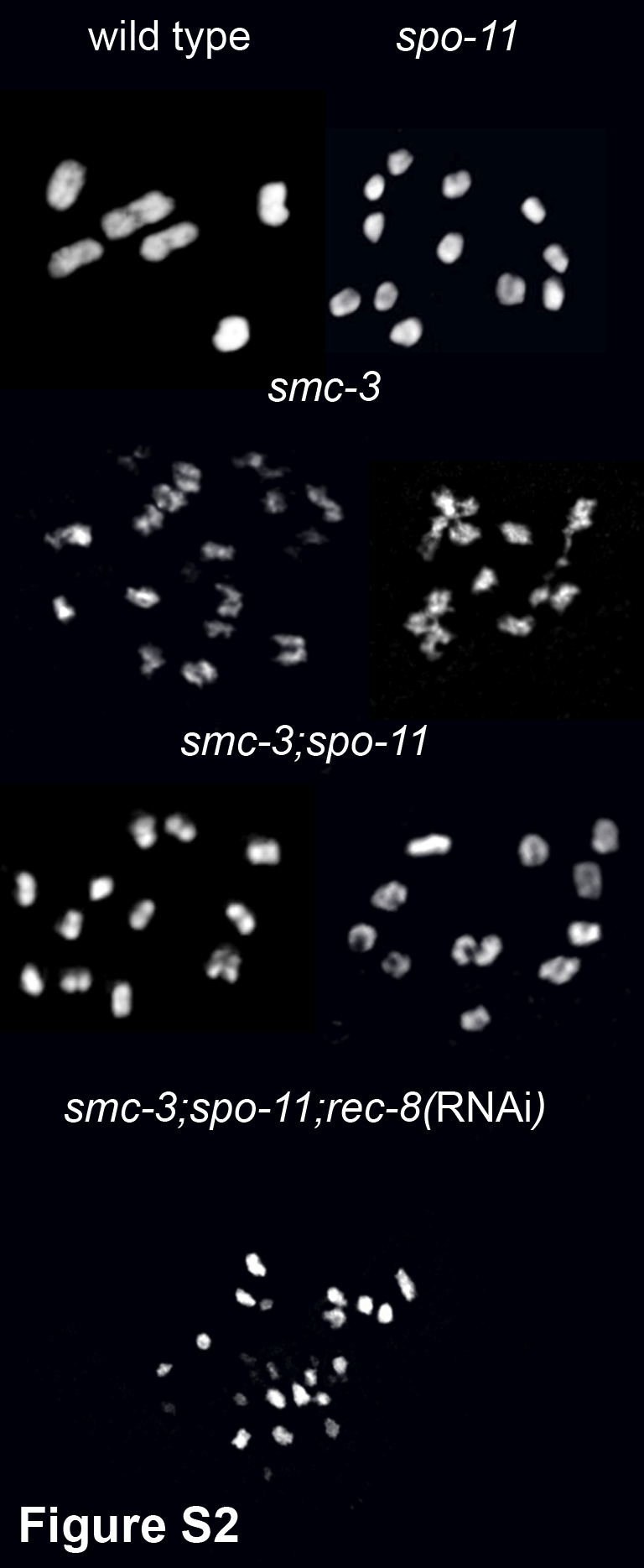

Supplement: Figure S2 — Meiotic cohesion is effective in smc-3 . Representative diakinesis of indicated genotypes (wild type, spo-11, smc-3, smc-3;spo-11, smc-3;spo-11;rec-8(RNAi). (TIFF) [file pone.0024799.s002.tiff]

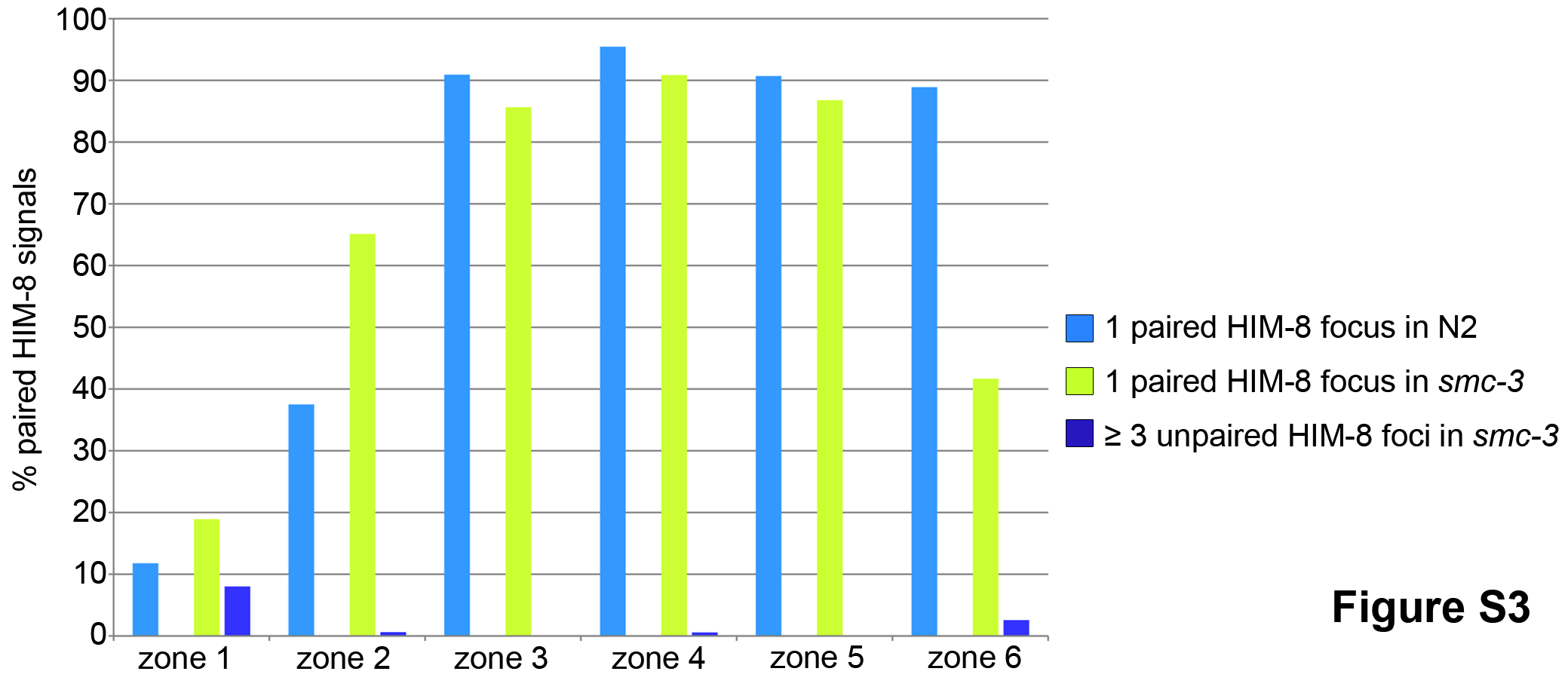

Supplement: Figure S3 — Rare mitotic defects in smc-3 mutants worms. Time course of HIM-8 pairing in wild type and smc-3 revealed the presence of 3 foci in the mitotic zone of smc-3 on rare occasions. (TIFF) [file pone.0024799.s003.tiff]

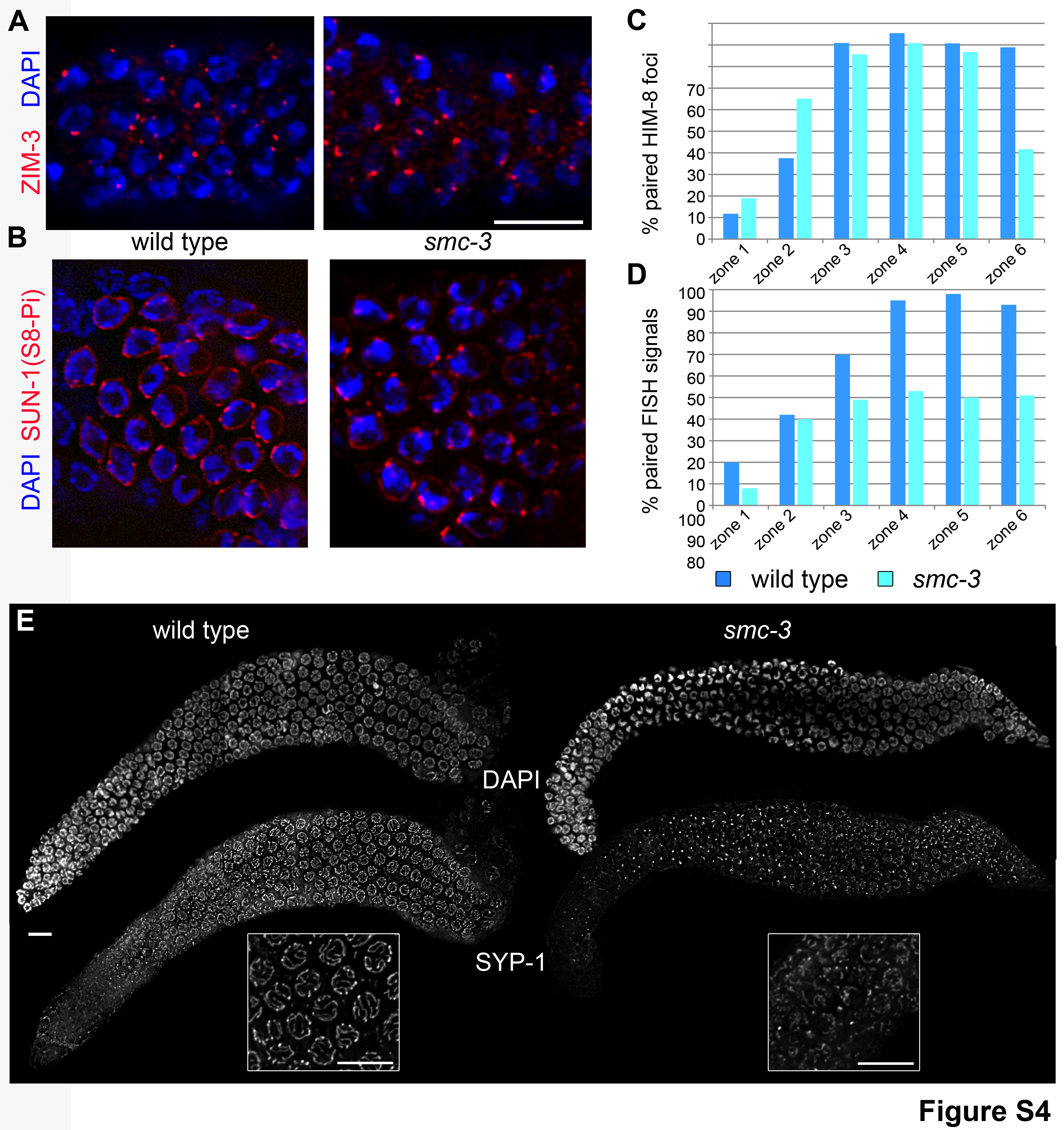

Supplement: Figure S4 — Proper loading of the PC protein ZIM-3 in smc-3 but defective synapsis. A. Immunostaining of the pairing center protein ZIM-3 in wild type and smc-3 (DAPI blue). B. Immunostaining of SUN-1S8Pi in wild type and smc-3; (DAPI blue). C. Time course for pairing of HIM-8 in wild type and smc-3. Gonads were subdivided into 6 zones of equal lengths. D. Time course for pairing with the 5S rDNA FISH probe (chromosome V) in wild type and smc-3. Gonads were subdivided into 6 zones of equal lengths. E. Immunostaining of SYP-1 in wild type and smc-3; pachytene nuclei enlarged in the inset; bar 10 µm. (TIFF) [file pone.0024799.s004.tiff]

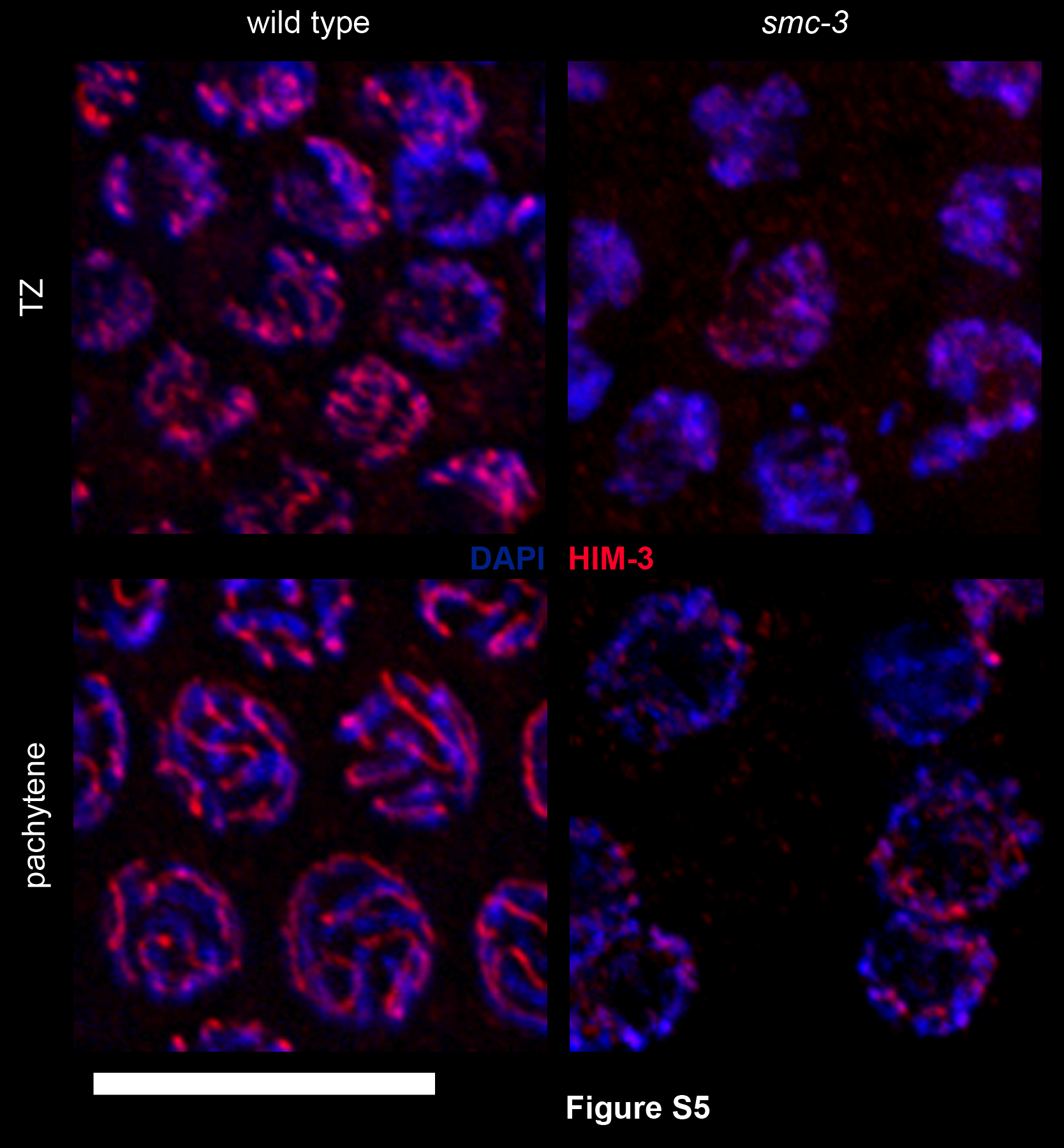

Supplement: Figure S5 — HIM-3 loading is strongly reduced in smc-3 . Immunostaining of HIM-3 (red) in wild type and smc-3; bar 10 µm. (TIFF) [file pone.0024799.s005.tiff]

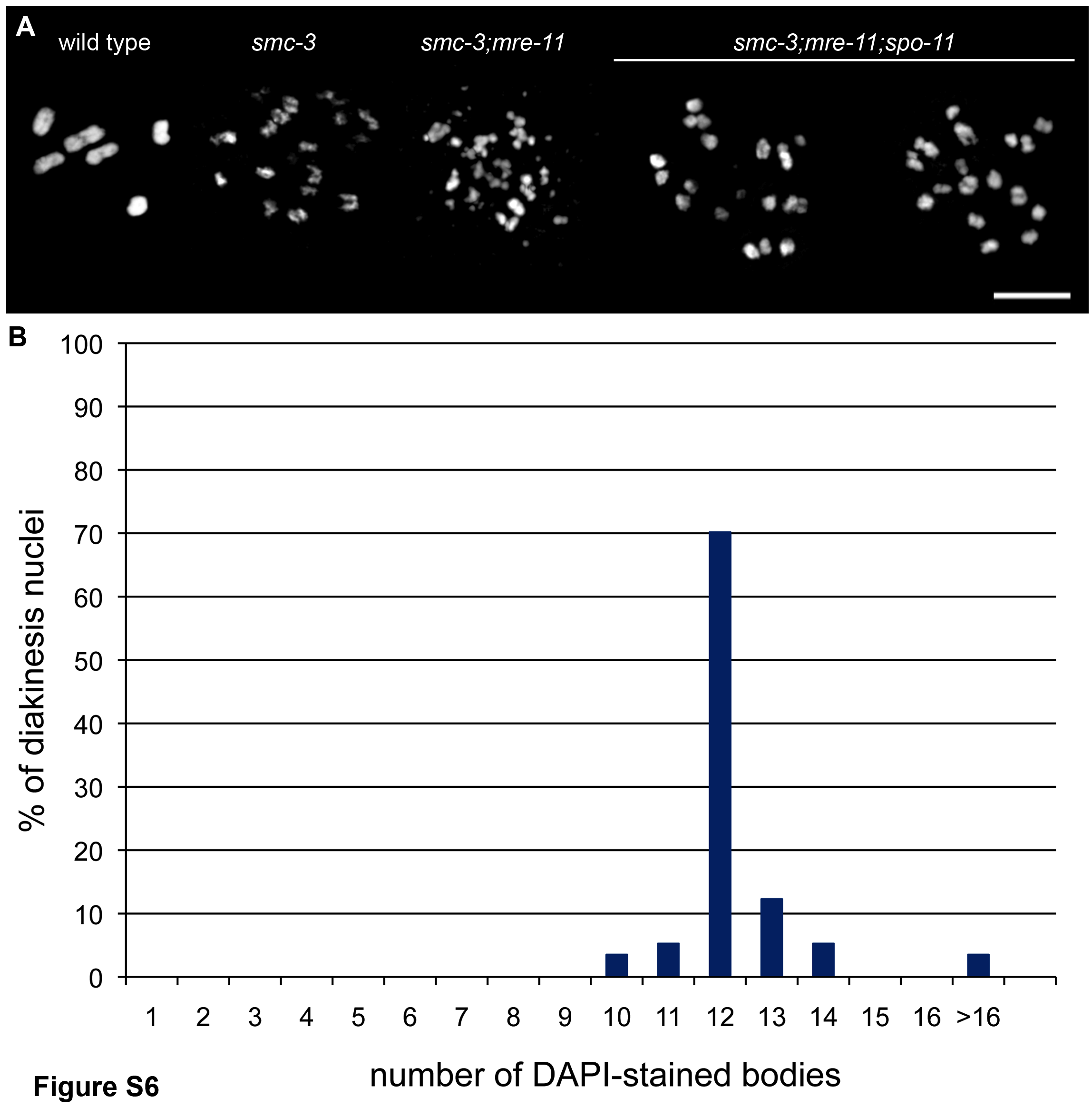

Supplement: Figure S6 — Absence of fragmentation in the triple mutant smc-3;spo-11;mre-11 . A. Representative diakinesis of the indicated genotypes (wild type, smc-3, smc-3;mre-11, smc-3;mre-11;spo-11). Bar 5 µm. B. Quantification of DAPI structures at diakinesis in smc-3;spo-11;mre-11. (TIFF) [file pone.0024799.s006.tiff]
