# Supplementary material for: A New Thermosensitive smc-3 Allele Reveals Involvement of Cohesin in Homologous Recombination in C. elegans
Source: PLoS One. 2011 Sep 21;6(9):e24799. doi: 10.1371/journal.pone.0024799 (PMC3177864; doi:10.1371/journal.pone.0024799)
Supplement: Text S1 — (DOC) [file pone.0024799.s013.doc]

**Supplemental information**

**Text S1**

**Stabilization of homolog pairing is compromised in *smc-3***

We wanted to test if *smc-3* mutants enter meiosis properly. In leptotene/zygotene, when homologous contacts are established in the wild type and vigorous chromosome movement takes place, Matefin/SUN-1 becomes phosphorylated at its N-terminus and forms aggregates at the nuclear periphery [1]. Homologous synapsis requires the action of chromosome-bound pairing center (PC)-binding proteins together with the integral nuclear envelope components Matefin/SUN-1 and ZYG-12, thereby linking chromosome ends to cytoplasmic motive forces [1,2,3].

Immunodetection of the PC protein ZIM-3 revealed that PCs were localized properly at the nuclear periphery in the *smc-3* mutant and homologous PCs were juxtaposed (Figure S4.A). Additionally, in *smc-3* gonads as in wild type, Matefin/SUN-1 aggregate formation and phosphorylation of SUN-1 Ser8 could be observed upon entry into meiosis (Figure S4.B). We therefore conclude that *smc-3* mutants are proficient in the loading of PC-binding proteins, the phosphorylation of SUN-1 and the formation of Matefin/SUN-1 aggregates and show no defect in progression of meiosis after meiotic entry.

We next investigated the pairing of the X chromosome and of chromosome V by scoring homologous associations of the PC protein HIM-8 [4] and of the 5SrDNA region (FISH), respectively. To this end, the hermaphrodite gonad was subdivided into 6 zones of equal length and for each zone the pairing status of the respective chromosome region was assessed. In *smc-3* mutants, pairing levels of the X chromosome were comparable to the wild type (Figure S4.C). Only in late pachytene (zone 6) pairing levels decreased significantly. Although pairing of the autosome V initiated in time (Figure S4.D), only about 50% of the chromosomes were paired in later stages of meiosis comparable to mutants lacking SC central element components [5,6,7].

These data demonstrate that in *smc-3* mutants the initial search process for the homologous partner started properly whereas initial pairing failed to be stabilized.

**Synapsis is compromised in *smc-3***

The observed failure to stabilize initial pairing led us to examine the polymerization of the central element of the SC. The integrity of the meiotic chromosome axis is a prerequisite for synapsis [6,8,9,10] and the formation of SUN-1 aggregates [1,11]. In the wild type, the transversal filament protein SYP-1 is first detectable in the transition zone as foci and eventually as short linear structures (Figure S4.E) and is fully polymerized along the entire length of paired homologous chromosomes in pachytene (Figure S4.E, left inset).

In *smc-3* gonads SYP-1 was detectable in nuclei with crescent-shaped chromatin suggesting that it was timely expressed. However, the protein remained concentrated in aggregates (polycomplexes) adjacent to the DNA instead of forming foci and short linear structures as in the wild type. Only a subpopulation of SYP-1 associated with the chromatin where SYP-1 formed foci and very short stretches, which elongated as nuclei progressed through meiotic prophase I (Figure S4.E). As long as SYP-1 polycomplexes were present, the chromatin stayed in a clustered configuration, a characteristic feature seen in SC mutants. Full SYP-1 polymerization between bivalents is accompanied by chromatin redistribution [5,6,7]. *smc-3* mutants displayed a prolonged zone of nuclei exhibiting a clustered configuration of chromatin (Figure S4.A).

In accordance with previous observations, the reduced loading of chromosome axis components in *smc-3* mutants affected the polymerization of the SC [12]. Indeed HIM-3 loading was strongly reduced in *smc-3* mutants (Figure S5) however to an extent possibly still permitting the loading of ZIM-3 and SUN-1 phosphorylation.

We conclude that the observed impaired stabilization of pairing was due to a failure in complete SC formation.

**Supplemental references**

1. Penkner A, Fridkin A, Gloggnitzer J, Baudrimont A, Machacek T, et al. (2009) Meiotic Chromosome Homology Search Involves Modifications of the Nuclear Envelope Protein Matefin/SUN-1. Cell 139: 920-933.

2. Penkner A, Tang L, Novatchkova M, Ladurner M, Fridkin A, et al. (2007) The Nuclear Envelope Protein Matefin/SUN-1 Is Required for Homologous Pairing in C. elegans Meiosis. Dev Cell. pp. 873-885.

3. Sato A, Isaac B, Phillips C, Rillo R, Carlton P, et al. (2009) Cytoskeletal Forces Span the Nuclear Envelope to Coordinate Meiotic Chromosome Pairing and Synapsis. Cell 139: 907-919.

4. Phillips CM, Wong C, Bhalla N, Carlton PM, Weiser P, et al. (2005) HIM-8 binds to the X chromosome pairing center and mediates chromosome-specific meiotic synapsis. Cell. pp. 1051-1063.

5. MacQueen AJ, Colaiácovo MP, McDonald K, Villeneuve AM (2002) Synapsis-dependent and -independent mechanisms stabilize homolog pairing during meiotic prophase in C. elegans. Genes Dev. pp. 2428-2442.

6. Colaiácovo MP, MacQueen AJ, Martinez-Perez E, McDonald K, Adamo A, et al. (2003) Synaptonemal complex assembly in C. elegans is dispensable for loading strand-exchange proteins but critical for proper completion of recombination. Dev Cell. pp. 463-474.

7. Smolikov S, Eizinger A, Hurlburt A, Rogers E, Villeneuve AM, et al. (2007) Synapsis-defective mutants reveal a correlation between chromosome conformation and the mode of double-strand break repair during Caenorhabditis elegans meiosis. Genetics. pp. 2027-2033.

8. Couteau F, Nabeshima K, Villeneuve A, Zetka M (2004) A component of C. elegans meiotic chromosome axes at the interface of homolog alignment, synapsis, nuclear reorganization, and recombination. Curr Biol. pp. 585-592.

9. Couteau F, Zetka M (2005) HTP-1 coordinates synaptonemal complex assembly with homolog alignment during meiosis in C. elegans. Genes Dev. pp. 2744-2756.

10. Martinez-Perez E, Villeneuve AM (2005) HTP-1-dependent constraints coordinate homolog pairing and synapsis and promote chiasma formation during C. elegans meiosis. Genes Dev. pp. 2727-2743.

11. Baudrimont A, Penkner A, Woglar A, Machacek T, Wegrostek C, et al. (2011) Leptotene/Zygotene Chromosome Movement Via the SUN/KASH Protein Bridge in *Caenorhabditis elegans*. PLoS Genet 6: e1001219.

12. Severson AF, Ling L, van Zuylen V, Meyer BJ (2009) The axial element protein HTP-3 promotes cohesin loading and meiotic axis assembly in C. elegans to implement the meiotic program of chromosome segregation. Genes Dev. pp. 1763-1778.
